# Congenital Vault Depression in an Infant Requiring Manual Pelvis Disengagement During Delivery

**DOI:** 10.7759/cureus.108134

**Published:** 2026-05-02

**Authors:** Alexander H Jin, Hannah M Brink, Glenda Weidendorf, Andrew J Groberg

**Affiliations:** 1 Pediatrics, Brooke Army Medical Center, San Antonio, USA; 2 Neonatology, Brooke Army Medical Center, San Antonio, USA

**Keywords:** birth injuries, caesarean delivery, fetal development, intra-uterine environment, pediatric skull fracture

## Abstract

Congenital vault depression (CVD) is a rare neonatal cranial finding that typically results from chronic intrauterine pressure and remodeling rather than acute delivery trauma. To the best of our knowledge, this report is the first that describes CVD in a term infant delivered via Caesarean section requiring manual pelvic disengagement due to a significantly impacted fetal head. Upon delivery, a 4 x 3 cm depression was noted in the right temporoparietal bone; however, the infant remained neurologically intact, and imaging via X-ray and computed tomography confirmed a concave deformity without evidence of fracture or intracranial injury. While neonatal skull depressions are typically attributed to either obstetric trauma or faulty fetal packing, another term for CVD, this report describes such a deformity occurring in the context of the manual pelvic disengagement maneuver. Managed conservatively through watchful waiting rather than surgical intervention, the infant demonstrated complete resolution of the skull depression by four months of age, supporting the efficacy of non-surgical management for asymptomatic presentations of suspected CVD.

## Introduction

In the immediate period after the delivery of a newborn infant, there are a variety of well-described cranial abnormalities that may be observed arising either from the birthing process or from intrauterine environmental factors, such as caput succedaneum, benign cranial molding, cephalohematoma, or subgaleal hemorrhage [1]. An uncommon, less well-described newborn cranial finding is a concave depression in the skull, the etiology of which is presumed to be due to either a depressed skull fracture (DSF) sustained during delivery or a congenital vault depression (CVD), also known as faulty fetal packing or intrauterine calvarial remodeling. CVD presents with an estimated frequency of approximately one in 10,000 live births [2], while neonatal skull fracture presents with an estimated incidence of approximately three in 10,000 births [3].

Skull fractures sustained at the time of delivery are most commonly caused by trauma from instrumentation or by pressure applied to the fetal head during challenging deliveries [3]. Although a CVD shares clinical and imaging features similar to those of a DSF, it is a distinct entity in that it develops in utero in the absence of overt or acute cranial trauma. It is thought to occur when either a fetal body part (e.g., fist) or maternal anatomical feature (e.g., uterine fibroid) places chronic pressure on the convex curvature of the skull and induces the fetal skull to form and shape in a concave depression around the impinging object [4]. There is scarce literature describing the presentation of this phenomenon, and distinguishing CVD from a skull fracture relies mainly upon clinical history and presentation. CVD and delivery-induced skull fractures both typically resolve by four to six months of age without the need for intervention [5]; however, some literature has explored neurosurgical management with techniques such as vacuum-assisted closed reduction to correct the depression [6,7].

In the case presented here, use of a vaginal hand for manual pelvis disengagement of the fetus was required at the time of delivery via low transverse Caesarean section (C-section). Vaginal hand is a technique required when the impacted fetal head has formed a vacuum seal with the engaged maternal pelvis, often after a trial of labor with deep descent into the pelvis, requiring an assistant to place a hand within the vagina to manually release the fetal head from the pelvis in order to be safely extracted via C-section [8]. Thus, this report describes a case of a skull depression in a term infant delivered via C-section without instrumentation, but with manual pelvis disengagement required at the time of delivery.

## Case presentation

A 26-year-old G2P0 pregnant woman presented at 39 weeks and five days for induction of labor due to newly diagnosed gestational hypertension. She had unremarkable maternal labs with low-risk genetic screening to include low-risk MaterniT21 (Labcorp Holdings Inc., Burlington, North Carolina, United States), negative alpha fetal protein, and negative testing for spinal muscular atrophy and cystic fibrosis. She was otherwise in a normal state of health, without any noted trauma during her pregnancy with a non-consanguineous partner. Maternal sexually transmitted infection (STI) testing was negative. Maternal vitamin D level was not collected. A fetal ultrasound at 20 weeks of gestation had confirmed unremarkable development and morphology.

Labor was initiated with oral misoprostol and an inserted Foley balloon. At 6 cm dilation, membranes were artificially ruptured, and fluid was noted to be colored with thin meconium. Although the mother was initially progressing without signs of fetal distress, approximately eight hours after administration of misoprostol, the fetus experienced a prolonged, seven-minute deceleration to a heart rate in the 80s, with improvement only to the low 100s. Thus, the decision was made to proceed urgently to C-section due to non-reassuring fetal heart tones in the concomitant setting of failure to progress. At the time of the decision to proceed to C-section, the fetus was engaged in the pelvis at 6 cm cervical dilation, 75% effacement, and -1 cm fetal station.

During delivery, no forceps or vacuum were used. However, due to a significantly impacted fetal head, there was a tight seal between the infant’s head and the mother’s bony pelvis during the first attempt at fetal extraction, a complication that can be present in as many as one in 10 emergent C-section deliveries [8]. Thus, a manual pelvic disengagement was performed to disrupt the seal and to elevate the fetal head toward the hysterotomy, a maneuver that is an accepted standard of care during similar instances of fetal head impaction against the pubic symphysis while attempting a C-section [8], though no significant data exists surrounding the complication rate of this maneuver. This maneuver was successful in allowing the infant to be delivered through the hysterotomy without difficulty.

Following delivery, the infant had appropriate initial tone and was spontaneously moving all extremities; however, given a weak, intermittent cry, cord clamping was not delayed, and the cord was cut at 30 seconds. The infant subsequently had a vigorous cry with warm/dry/stimulation on the infant warmer, and APGAR scores were 7 and 9 at one and five minutes, respectively. Birth measurements were notable for weight, small for gestational age (2,550 grams), but otherwise appropriate for gestational age length (49.3 cm) and head circumference (33.6 cm).

Initial physical exam was notable for a depression in the right temporoparietal bone measuring approximately 4 x 3 cm in area and 0.5 cm in depth. There was no noted crepitus, edema, ecchymosis, bogginess, or palpable instability of the bony structures in the area. A firm base was present underneath the lesion, suggesting no absence of bone. Otherwise, the skull appeared normocephalic with appropriately sized fontanelles and appropriately positioned sutures. The patient appeared neurologically appropriate, with normal and symmetric tone and intact neonatal reflexes bilaterally.

Further serial neurologic exams in the hours and days following delivery showed no acute changes in exam findings. Of note, there was also no development of any ecchymosis or swelling over the area of depressed skull at any point during his approximately 60-hour birth admission, nor at his initial follow-up (~48 hours after discharge). There were no other dysmorphic features noted, and the remainder of his physical exam was unremarkable.

**Figure 1:**
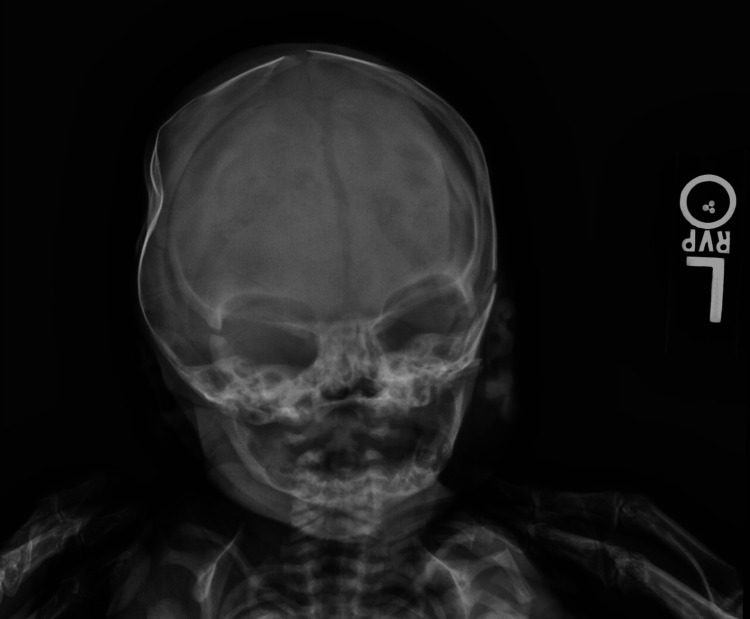
Head X-ray of the neonate revealing concave deformity of the right parietal calvarium with concomitant diastasis of the sagittal suture.

Initial imaging included a two-view head X-ray (Figure 1) completed immediately after delivery that revealed a concave deformity of the right parietal calvarium with concomitant diastasis of the sagittal suture. Bony structures underneath the deformity were intact, without evidence of fracture, despite the concave structure. Mild soft tissue swelling was also noted overlying the concavity. 

**Figure 2:**
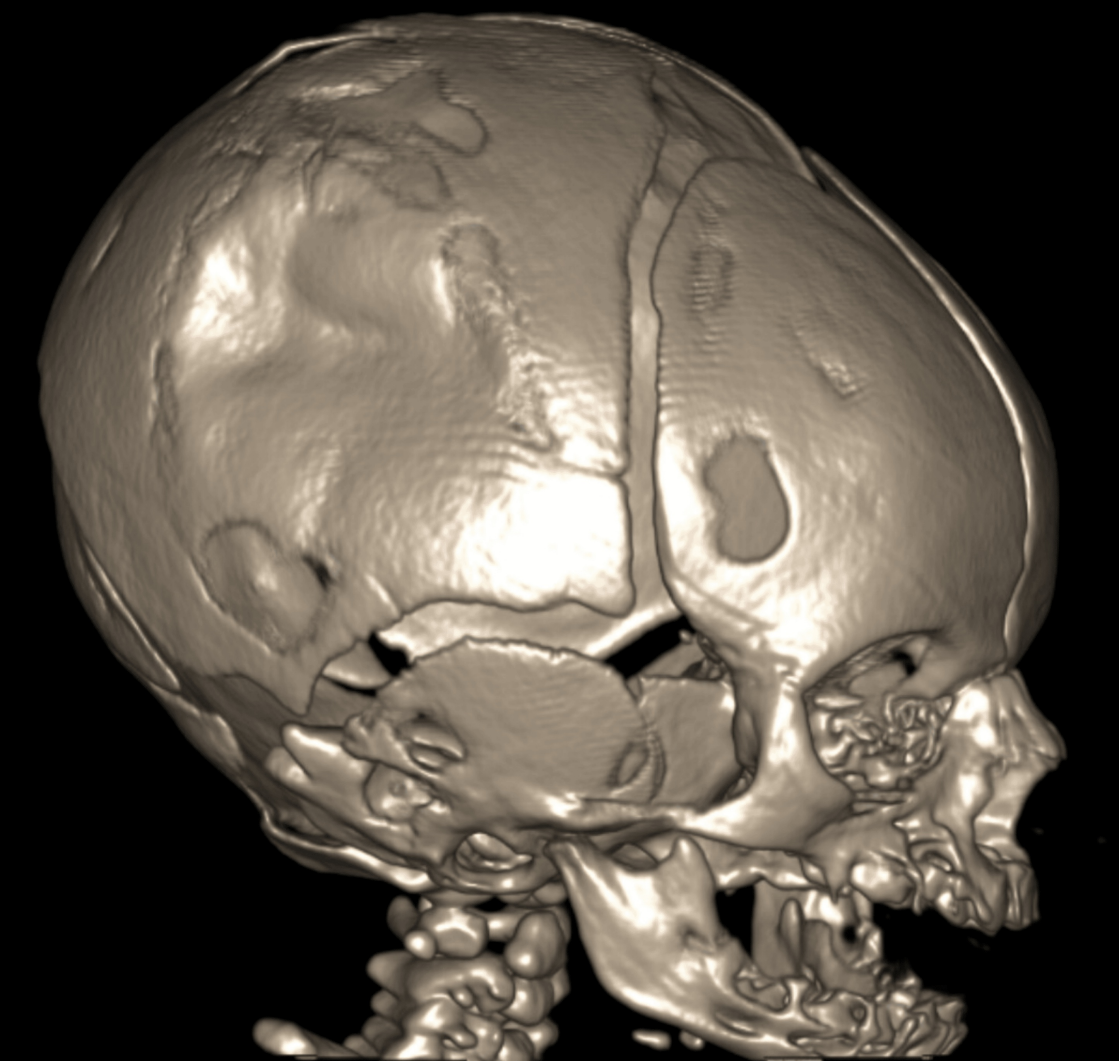
CT head with skull reconstruction showing right parietal bone depression with no visualized fracture lucency or acute hemorrhage, with intact gray-white matter differentiation without mass effect or midline shift.

A non-urgent head computed tomography (CT) was obtained on day two of admission to further characterize the depression, at which time there was a low clinical concern for acute intracranial injury. The CT head (Figure 2) re-demonstrated a depression of the right parietal bone with now-resolved scalp soft tissue swelling. There was no visualized fracture lucency, no acute hemorrhage, and intact gray-white matter differentiation without mass effect or midline shift. Expert opinion was sought, and a pediatric neuroradiologist who reviewed the images stated that both the head X-ray and CT head did not have radiologic features consistent with a true fracture.

The infant did not present for a two-month well visit, but at the four-month well visit, the skull depression was noted to have completely resolved. The infant was healthy and developing appropriately at that time, with head circumference tracking appropriately along the 57th percentile. At the 18-month well visit, the patient continued to maintain an appropriate head circumference at the 58th percentile, and the 24-month well visit noted no significant developmental abnormalities or abnormal neurological findings. 

## Discussion

Skull depressions are rare in newborns [9], and the management remains somewhat unclear. As previously mentioned, some appear to be the result of either obstetric trauma involving instrumentation and/or manual manipulation of the head leading to an acute DSF [2], while others are due to congenital vault depression caused by pressure on the fetal skull by maternal anatomy (i.e. bony prominences or uterine masses) or fetal body parts that are chronically impacted against one part of its skull [4,10]. Distinguishing between these precipitating factors is challenging, given their similar clinical and radiologic findings. The only described non-specific finding for DSF is indentation marks that fit the marks of the forceps blades [2]. Other risk factors for skull fracture include abdominal trauma during pregnancy, congenital disorders of osteogenesis, or Ehlers-Danlos syndrome [11]. 

For the neonate in the current report, there was no instrumentation used, and no other clinical manifestations or history consistent with any other known risk factors predisposing to fractures. However, the clinical picture is complicated by the use of a vaginal hand to disengage the fetal head from the pelvis. To date, the occurrence of a skull depression in the context of this maneuver has not been described in the literature, though there are described cases of manual manipulation of the skull during difficult vaginal deliveries leading to depressed skull fractures [9].

The presumed diagnosis for this infant is a congenital vault depression, given the absence of instrumentation during delivery and the presence of a skull concavity noted at birth that did not present with or develop any typical signs of fracture, including overlying soft tissue swelling, ecchymosis, crepitus, cortical defect on CT, or associated intracranial injury [12]. However, there is admittedly a paucity of physical exam descriptions of the characteristics of the skin overlying a skull DSF.

The prognosis for cases of infants with skull depressions at delivery is varied, and the severity of outcomes appears to be directly related to whether or not instrumentation was used during delivery. A retrospective, case-control analysis of 68 infants with depressed skull fractures noted on delivery found that of the 18 infants with no documented use of instrumentation to augment the delivery process, none of them suffered any significant neurological complications or indications for emergent neurosurgery, whereas 30% of the 50 infants for whom instrumentation was required did have intracranial lesions that required urgent or emergent neurosurgical intervention [13]. A growing body of research has suggested that in the absence of neurological deficit, symptoms of intracranial injury (e.g. seizure), or radiological evidence of intracranial findings requiring neurosurgical correction, conservative management can be safe and effective for these infants as their fractures self-resolve with no difference in outcomes between surgically and non-surgically treated patients in terms of long-term outcomes including development of seizures and/or epilepsy, neurological dysfunction, or cosmetic appearance [14]. Additionally, these symptoms will typically manifest within the time of hospitalization, immediately following delivery [9]; thus, if there have been no concerns for clinical stability prior to discharge in the American Academy of Pediatrics (AAP)-recommended 48-72 hours [15], there is a low likelihood of clinical deterioration following discharge.

Given that our patient remained neurologically appropriate with serial exams and had no radiological evidence of findings requiring neurosurgical intervention throughout the duration of his hospitalization, he was conservatively managed with watchful waiting and close follow-up, allowing him to remain with his parents in dyad post-partum care and avoid prolonged separation. Finally, he was noted to have appropriate interval resolution of his CVD at his four-month well visit.

Although more data align with CVD for our patient, a fracture cannot be completely ruled out, given the lack of clear diagnostic criteria for both presentations. Further investigation is needed into differentiating between the clinical findings and imaging of CVD and neonatal depressed skull fracture. This case presents a rare occurrence of skull depression in a newborn and another mechanism for neonatal skull depression. Limitations of this case include the inability to identify the causality of the underlying lesion and difficulty generalizing the findings to other similar obstetric deliveries. 

## Conclusions

This was a case of likely CVD, hypothesized to be due to intrauterine calvarial remodeling in a term infant delivered via C-section, for whom manual disengagement of fetal head from the pelvis was required at the time of delivery. This appears to be the first described case of skull depression in a delivery requiring the aforementioned maneuver. Skull depressions in neonates are rare and are typically caused either by intrauterine calvarial remodeling or obstetric trauma. No management guidelines exist, but conservative intervention was sufficient for interval resolution of the skull depression in this patient. Long-term prognosis in an initially asymptomatic patient, as in our case, appears to be equivalent to that of those without any significant calvarial deformities noted on delivery.
